# The value of serum Krebs von den lungen-6 as a diagnostic marker in connective tissue disease associated with interstitial lung disease

**DOI:** 10.1186/s12890-019-1043-z

**Published:** 2020-01-08

**Authors:** Hua Ma, Junhui Lu, Yuanyuan Song, Huixuan Wang, Songlou Yin

**Affiliations:** 1 Department of Rheumatology and Immunology, Affiliated Hospital of Xuzhou Medical University, No. 99 Huaihai West Road, Xuzhou, 221002 China; 2 Department of Rheumatology and Immunology, Huai’an Second People’s Hospital, Huai’an, 223002 China

**Keywords:** Connective tissue disease, Interstitial lung disease, Connective tissue disease associated with interstitial lung disease, Krebs von den lungen-6

## Abstract

**Objectives:**

The purpose of this study was to evaluate the value of serum krebs von den lungen-6 (KL-6) level as a diagnostic indicator for connective tissue disease associated with interstitial lung disease (CTD-ILD).

**Methods:**

One hundred fifty five patients with newly diagnosed CTD in our hospital were enrolled and divided into two groups by their ILD manifestations, the CTD-ILD group and the CTD group. In parallel, 61 patients with pulmonary infection and 60 cases of healthy subjects were also enrolled into the study. The difference of serum KL-6 level among the four groups were compared. In CTD-ILD group, carbon monoxide diffusing capacity (DLCo) and high-resolution computed tomography (HRCT) of lung were also tested. The serum KL-6 level of 32 patients from the CTD-ILD group who received cyclophosphamide (CTX) pulse therapy were sampled and measured, by enzyme linked immunosorbent assay (ELISA), at three time points: before treatment, 3 months after treatment and 6 months after treatment.

**Results:**

The serum KL-6 level in the CTD-ILD group (1004.9 (676.41738.1) IU/ml) is significantly higher than three other groups (*χ*^*2*^ = 72.29, *P <* 0.001). In the CTD-ILD group the level of serum KL-6 was positively correlated with disease severity on HRCT (*r* = 0.75, *P* <  0.001), while was negatively correlated with DLCo (*r* = − 0.50, *P* <  0.001). In 32 patients who received CTX pulse therapy, the level of serum KL-6 was gradually decreased in 20 cases whose lesions were absorbed within 6 months (*F* = 13.67, *P* <  0.001), whereas it remained unchanged in the rest of 12 patients (*Z* = -1.328, *P* = 0.198).

**Conclusions:**

Serum KL-6 level can potentially serve as a diagnostic marker for CTD-ILD and be utilized to evaluate the effectiveness of CTX pulse therapy.

## Introduction

The connective tissue diseases (CTD) are a collective series of immune-mediated pathologies and systemic disorders affecting multiple connective tissues, including chronic inflammation of blood vessels [1]. The lung is rich in blood vessels, collagen and other connective tissues, and play important functions in immune, endocrine and metabolic regulations. Therefore, it often becomes the first affected organ of CTD. The ILD is a common complication of CTD and a key risk factor of mortality, which is often diagnosed at an advanced stage. Typically, the onset of ILD is insidious, with only subtle clinical symptoms [2, 3].

Currently, pulmonary function test (PFT) and high-resolution computed tomography (HRCT) are important diagnostic tools for the assessment of severity of CTD-ILD [4]. However, HRCT may occasionally cause lung damage, even though mostly rare [5]. As the pathological basis of ILD presents as diffuse alveolar inflammation and pulmonary interstitial fibrosis, the main pathophysiological process is resulted from the injuries and regeneration of alveolar type II epithelial cells [6]. In recent years, many studies suggested that a variety of cytokines associated with alveolar injuries may be useful for the diagnosis of CTD-ILD and may provide some valuable information for disease prognosis [7].

The Krebs von den Lungen-6 (KL-6) is a mucin-like glycoprotein mainly expressed on the type II alveolar epithelial cell surface [8]. The expression of KL-6 is significantly increased when the alveolar epithelium sustains injuries, and the KL-6 can be secreted into the bloodstream through the damaged alveolar basement membrane [9]. Thus, elevated serum KL-6 levels may indicate the onset of CTD in patients with ILD and may be used to predict prognosis [10, 11]. However, other studies showed that KL-6 was a poor prognostic factor for systemic sclerosis (SSc)-ILD [12, 13]_**.**_ Furthermore, the efficacy of KL-6 to assess the therapeutic effect remains understudied. The main goal of our study was not only to assess if the serum KL-6 level can be included into the clinical practice as a reliable diagnostic indicator for CTD-ILD patients, but also to evaluate possibility to use the value of KL-6 to evaluate the efficacy of CTX treatment on CTD-ILD patients.

## Methods

### Patient recruitment

A total of 155 patients with newly diagnosed CTD from January 2016 to June 2016 in the Affiliated Hospital of Xuzhou Medical University were recruited into this study and were divided into two groups according to their ILD manifestations. All patients were eligible for classification/diagnosis criteria of the corresponding disease. Sixty one cases of pulmonary infection, 60 cases of healthy subjects (who only underwent physical exams) were collected in the same period.

### Inclusion criteria

Patients were diagnosed as CTD-ILD according to the imaging changes based on HRCT: mesh shadows, solid shadows, cellular shadows, ground glass shadows, and nodular shadows [13, 14].

### Exclusion criteria

1. With other lung diseases such as tuberculosis, chronic obstructive pulmonary disease and pulmonary infection; 2. Other causes of pulmonary interstitial fibrosis, such as pneumoconiosis, radioactive pneumonia; 3. Severe organ dysfunction such as heart failure, renal failure, pulmonary hypertension; 4. Patients with malignant tumor; 5. Pregnant and lactating women.

### CTX pulse therapy

The efficacy of the CTX pulse therapy were determined by the following criteria [15]: [1] Invalid: dry cough or wheezing symptoms deteriorate; vital capacity (VC) or DLCo decreased more than 10% compared with baseline; progression of lung lesion range based on HRCT score [2]. Effective or stable: does not meet any of the above.

### Serum KL-6 measurements

Peripheral fasting blood samples were collected at early morning. The separation of the serum was performed at 3000 rpm for 15 min, and stored at − 80 °C. The serum KL-6 levels were detected by ELISA (Shanghai sailing Biological Technology Co. Ltd). Each measurement value for standard antigen and serum was an average value from two tested samples. In CTD-ILD group, 32 out of total 84 patients were treated with CTX pulse therapy. The assessment of serum KL-6 levels of these 32 patients at three time points, which were: before treatment, 3 months after treatment and 6 months after treatment.

### Pulmonary function tests

In parallel to the serum KL-6 level measurement, HRCT and PFT were performed on the 67 CTD-ILD patients.

The HRCT score: take the aorta arch margin, carina, 1 cm above the diaphragm, respectively, calculate the three layers of fibrosis accounted for the percentage of the corresponding lung field area. The sum of the scores from three layers is the HRCT score (Table 1). All HRCT images were analyzed by two thoracic radiologists.

**Table 1:** HRCT scores

Score	Involving the range (%)
0	Normal
1	1–25
2	26–50
3	51–75
4	> 75

HRCT score: take the aorta arch margin, carina, 1 cm above the diaphragm, respectively, calculate the three layers of fibrosis accounted for the percentage of the corresponding lung field area, 0 points: normal; 1 points: involving the range of 1–25%; 2 points: involving the range of 26–50%; 3 points: involving the range of 51–75%; 4 points: no more than 1% Involving a range of more than 75%

### Statistical analysis

The comparison between the two groups of measurement data used Student’s *t*-test in the normal distribution, Wilcoxon two sample test when the normal distribution was not used, and the normality in the three groups and above were analyzed by one-way ANOVA. The Kruskal-Wallis test was used for normality, and the SNK method was used after the comparison. KL-6 comparisons before and after treatment were performed using paired *t*-test or signed rank sum test. The count data was compared between the two groups using the *chi*-square test or the Fisher exact probability method. Logistic multivariate regression analysis was used to analyze the risk factors for ILD, and correlations between KL-6, HRCT, and DLCo were analyzed using Pearson correlation and Spearman correlation coefficient. The ROC curve of the ILD occurrence for each indicator is plotted, and the cutoff value is taken when the Youden index is the largest. Statistical analysis was performed using SAS 9.3. Use a two-sided test. The difference was considered statistically significant at *P* <  0.05.

## Results

### General information on study subjects

The patients enrolled included 204 females (73.9%) and 72 males (26.1%) aged average 53.13 ± 11.19 years old (from 14- to 89- year-old) (Table 2). From the enrolled CTD patients, 31 patients had rheumatoid arthritis (20.0%), 24 patients had Sjögren’s syndrome (15.48%), 29 patients had systemic lupus erythematosus (18.06%), 25 patients had idiopathic inflammatory myopathy (16.13%), 26 patients had systemic sclerosis (16.77%), 7 patients had mixed connective tissue disease (4.52%), 3 patients had overlap syndrome (1.94%), and 10 patients had vasculitis (6.45%). Finally, the overall incidence of ILD was 54.19% (Table 3).

**Table 2:** The age and gender of each patient group

Characteristics	CTD-ILD *n* = 84	CTD *n* = 71	Pulmonary infection *n* = 61	Healty controls *n* = 60	p
Gender, n (%)					0.342
Female	64 (76.19)	56 (78.87)	40 (65.57)	44 (73.33)	
Male	20 (23.81)	15 (21.13)	21 (34.43)	16 (26.67)	
Age (years), mean ± SD	51.2 ± 13.75	53.8 ± 11.58	54.6 ± 12.89	52.9 ± 15.67	0.07

**Table 3:** Basic characteristics of patients in each of the four groups

Characteristics	CTD-ILD, n = 84	CTD, n = 71	p
RA,n(%)	17 (20.24)	14 (19.72)	0.936
SS,n(%)	12 (14.29)	12 (16.90)	0.654
SLE,n(%)	13 (15.48)	16 (22.54)	0.262
IIM,n(%)	14 (16.67)	11 (15.49)	0.843
SSc,n(%)	17 (20.24)	9 (12.68)	0.209
MCTD,n(%)	4 (4.76)	3 (4.23)	1.000
Vasculitis,n(%)	5 (5.95)	5 (7.04)	1.000
OS,n(%)	2 (2.38)	1 (1.41)	1.000
Rash,n(%)	14 (16.67)	19 (26.76)	0.126
Raynaud’s phenomenon,n(%)	38 (45.24)	4 (5.63)	< 0.001
Arthritis,n(%)	32 (38.10)	39 (54.93)	0.036
Fever,n(%)n)	13 (15.48)	13 (18.31)	0.638
ANA,n(%)	66 (78.57	43 (60.56)	0.014
RF,n(%)	38 (45.24)	37 (52.11)	0.393
ESR (mm/H), median (IQR)	40 (21,70)	42 (21,60)	0.994
CRP (mg/L), median (IQR)	4.77 (2.28,18)	8.31 (2.52,31.8	0.143
LDH(U/L), median (IQR)	235 (185,333)	202 (169,268)	0.039
IgG(g/L), median (IQR)	17.35 (15.05,21.7)	16.1 (12.6,20.1)	0.098
C3(g/L), median (IQR)	0.93 (0.8,1.1)	0.93 (0.61,1.2)	0.849
C4(g/L), median (IQR)	0.18 (0.14,0.23)	0.19 (0.12,0.27)	0.763
WBC(m × 10^9^/L), median (IQR)	5.8 (4.4,9.1)	6.9 (4.1,8.6)	0.994
N(×10^9^/L), median (IQR)	3.58 (2.53,6.4)	4.03 (2.55,5.91)	0.986
Hb(g/L), median (IQR)	119.5 (110,135)	117 (103,128)	0.115
PLT(×10^9^/L), median (IQR)	224 (169.5299)	239 (155,305)	0.839

*RA* Rheumatoid arthritis, *SS* Sjogrensyndrome, *SLE* Systemic lupus erythematosus, *IIM* Idiopathic inflammatory myopathy, *SSc* Systemic sclerosis, *MCTD* Mixed connective tissue disease, *OS* Overlap syndrome, *CTD* Connective tissue disease, *CTD-ILD* Interstitial lung disease associated with connective tissue disease

In the CTD-ILD group, all patients had received HRCT scanning and 67 patients had received pulmonary function test (PFT). Also, 5 patients did not meet the criteria for PFT due to critical illness, and 12 patients refused to accept the inspection.

### The serum KL-6 level varies in different groups

The serum KL-6 level in CTD-ILD group (medium level, 1004.95 IU/ml) was higher than CTD group (medium level, 467.99 IU/ml). We also found that the serum KL-6 level in CTD-ILD patients was significantly higher than those in patients with pulmonary infection and normal physical examination group (Table 4).

**Table 4:** The serum KL-6 level from the four different groups of patients

Groups	KL-6 level (IU/ml) Median (IQR)	p
CTD-ILD	1004.9 (676.41738.1)	< 0.001^a^
CTD	468.0 (272.61005.9)	< 0.001^b^
Pulmonary infection	526.4 (439.7, 670.0)	< 0.001^c^
Normal	452.5 (338.3, 550.0)	< 0.001^d^

*CTD* Connective tissue disease, *CTD-ILD* Interstitial lung disease associated with connective tissue disease. Note: ^a^comparing among 4 groups; ^b-d^each compared to CTD-ILD group

### Correlations between the serum KL-6 level and the severity of CTD-ILD disease

**Fig. 1:**
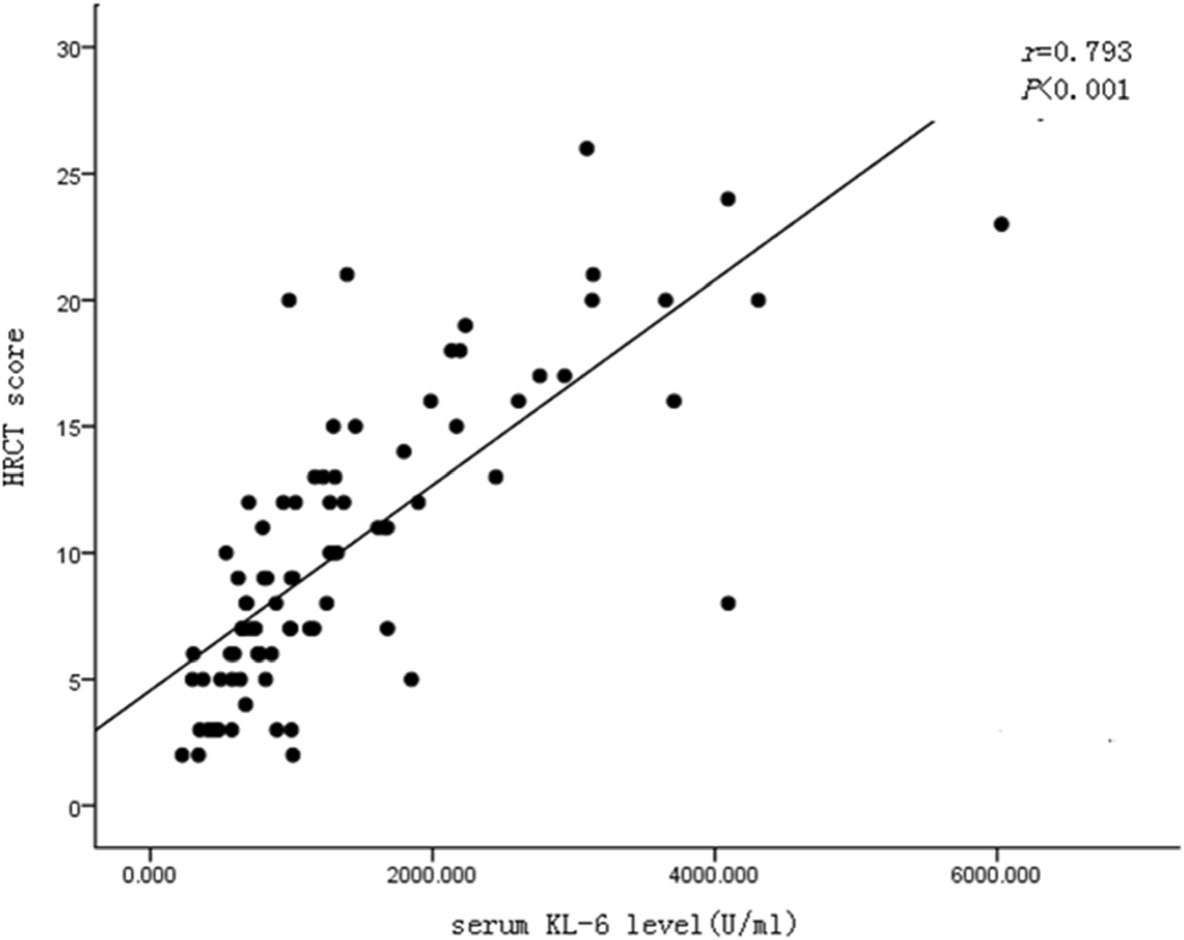
The correlation between serum KL-6 level and HRCT scores. The level of serum KL-6 was positively correlated with HRCT scores (*r* = 0.75, *P* <  0.001)

**Fig. 2:**
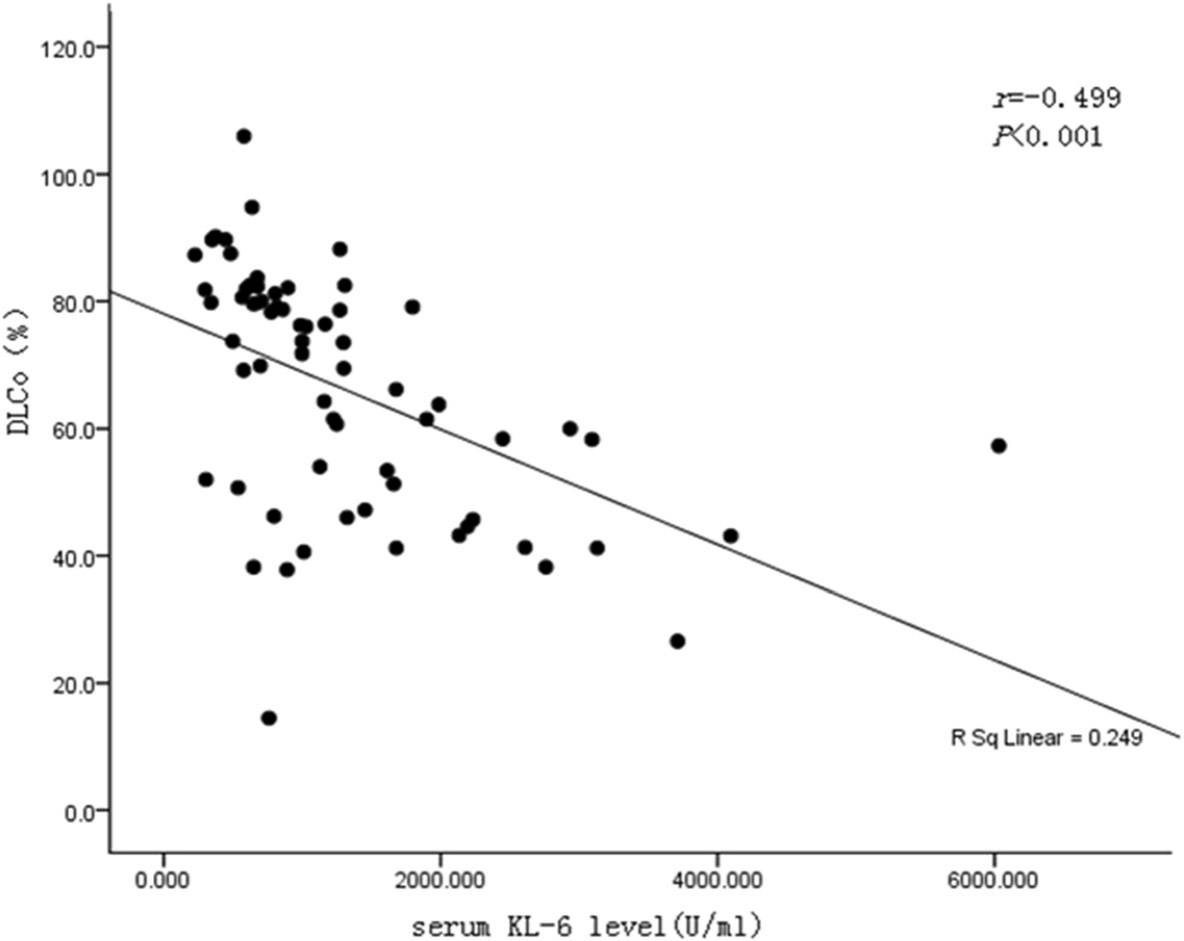
The relationship between serum KL-6 levels and DLCo. The level of serum KL-6 was negatively correlated with DLCo (*r* = − 0.50, *P* <  0.001)

The HRCT scanning and DLCo are used to evaluate the extent and severity of CTD-ILD disease. The higher HRCT scores indicate the wider extent of CTD-ILD affected tissues, while the lower DLCo scores indicate the more severe CTD-ILD disease states_**.**_ The level of serum KL-6 was positively correlated with HRCT scores (*r* = 0.75, *P* <  0.001), and was negatively correlated with DLCo (*r* = − 0.50, *P* <  0.001) (Figs. 1 and 2), suggesting that serum KL-6 levels may be used as an auxiliary indicator for HRCT and DLCo for patient assessment.

### The serum KL-6 level correlates with the prognosis after CTX treatment

**Fig. 3:**
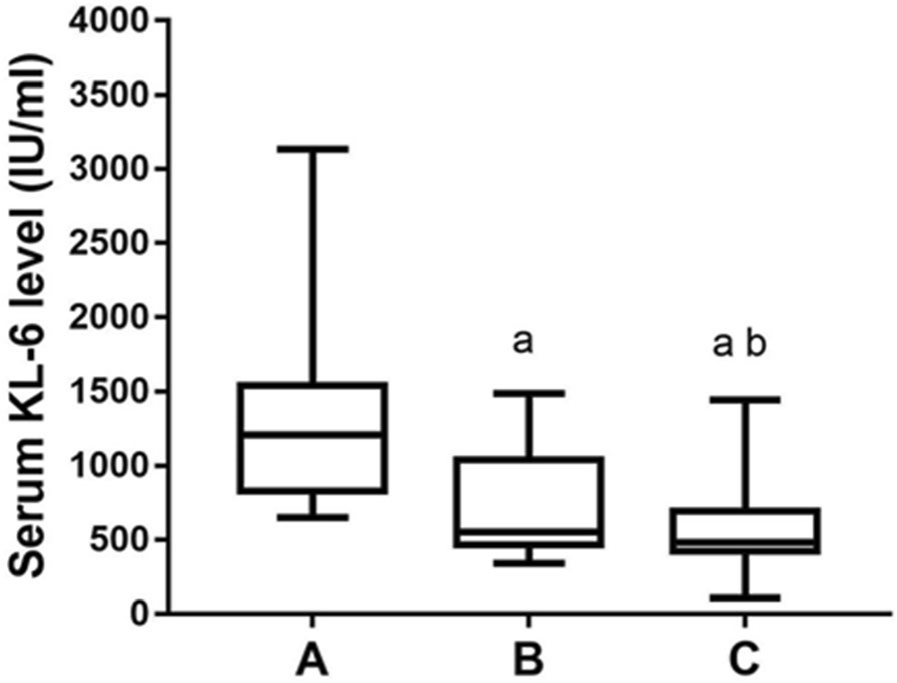
The changes of serum KL-6 level in 20 patients with improved condition after CTX therapy. The serum KL-6 levels gradually decreased over time in the patients with disease remission diagnosis. A: before treatment, B: 3 months after treatment, C: 6 months after treatment. a, compared with before treatment *p* <  0.05; b, compared with 3 months after treatment *p* < 0.05

**Fig. 4:**
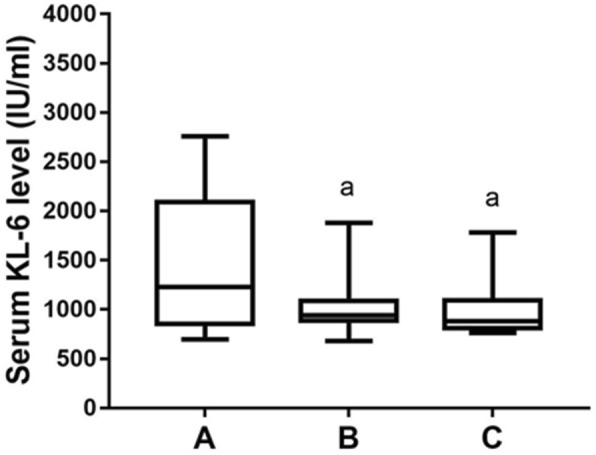
The changes of serum KL-6 level in 12 patients after CTX therapy. The serum level of KL-6 showed no significant changes between 3 months and 6 months after treatment in non-responsive patients. A: before treatment, B: 3 months after treatment, C: 6 months after treatment. a, compared with before treatment p < 0.05

After received CTX pulse therapy, all 32 patients received HRCT scanning and PFT at three time points to assess the effectiveness of their treatment. Overall there were 20 patients diagnosed as remission, and the rest of 12 patients were classified as non-remission. Within those 20 patients with disease remission diagnosis, their serum KL-6 levels gradually decreased over time (Fig. 3). However, high serum level of KL-6 persisted (> 1000 IU/ml) at all time points among non-remitters, even though whose disease condition did improve to some extents after treatment compared to pre-treatment (Fig. 4).

### The effectiveness of using serum KL-6 level measurement as a risk factor for ILD occurrence

**Fig. 5:**
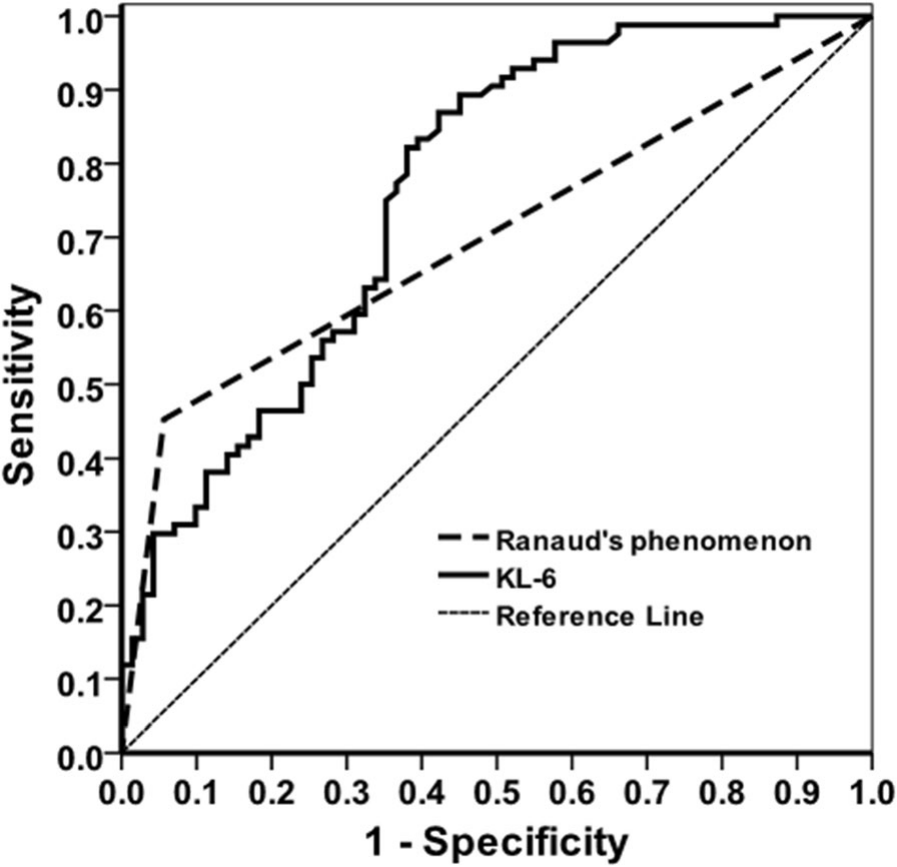
The ROC curve for serum KL-6 level for usefulness evaluation. The area under the curve is 0.81. When the cut-off value is set to 617.73 IU/ml, sensitivity (0.82) and specificity (0.72) reach the maximum, which is the best working point

Using logistic multivariate regression, we showed that among several patient characteristics, high serum KL-6 level and Raynaud’s phenomenon served as good indicators for the onset of CTD-ILD (Table 5). Upon further analysis, we showed that the area under the curve (AUC) of the receiver operating characteristic (ROC) curve from KL-6 is 0.76, higher than 0.69 from Raynaud’s phenomenon (Table 6 and Fig. 5), which suggested the serum KL-6 level indeed has a decent, if not better, predicting value as a risk factor for ILD occurrence.

**Table 5:** The risk factors for CTD-ILD occurrence

Model	Variables	Risk Factor	Parameter	OR	95%CI of OR		p
					Lower	Upper	
Model 1 ^a^			−1.24	–	–	–	–
	KL-6	Unit = 100	0.141	1.151	1.069	1.240	< 0.001
	LDH	Unit = 20	0.001	1.001	0.990	1.013	0.826
	IgG	Unit = 1	0.037	1.038	0.977	1.102	0.226
	Raynaud’s phenomenon	Yes VS. No	1.162	10.226	3.197	32.712	< 0.001
	Arthritis	Yes VS. No	−.317	0.531	0.234	1.207	0.131
	ANA	Yes VS. No	0.234	1.597	0.642	3.970	0.314
Model 2 ^b^			−.315				
	KL-6	Unit = 100	0.129	1.138	1.063	1.219	< 0.001
	Raynaud’s phenomenon	Yes VS. No	1.224	11.573	3.716	36.040	< 0.001

Note: ^a^Logistic multivariate regression was included as an independent variable for the variable of *p* < 0.100 and KL-6 in Tables 3 and 4.; ^b^Logistic multivariate regression using KL-6 and Raynaud’s phenomenon as independent variables

**Table 6:** The predicting values of CTD-ILD occurrence from KL-6 and Raynaud’s phenomenon

Source	AUC of ROC	95%CI of AUC		Sensitivity	Specificity	Positive predictive values	Negative predictive values	Cut-off valve
		Lower	Upper					
KL-6	0.762	0.687	0.838	0.821	0.620	0.684	0.776	617
Raynaud’s phenomenon	0.698	0.638	0.758	0.452	0.944	0.890	0.633	–

## Discussion

The ILD is an important manifestation of visceral involvement in patients with CTD, and often diagnosed at an advanced stage. About 15% of patients are affected with both CTD and ILD [16]. The CTD-ILD, which is caused by autoimmune responses in the alveolar epithelium with different degrees of inflammation and fibrosis. Early manifestations of CTD-ILD are exudative alveolitis, widely deposited late collagen fibers, resulting in irreversible changes in pulmonary fibrosis and pulmonary dysfunction [17]. The decline of lung function caused by the CTD-ILD directly leads to increased mortality in patients and impacts the prognosis of patients [18]. At present, CTD-ILD diagnosis mainly depends on HRCT and pulmonary function test, but these tests have some inherent limitations such as high cost, poor reproducibility, as well as the incompatibility for critically ill patients.

The KL-6 is a glycoprotein antigen expressed on alveolar type II epithelial cells and bronchial epithelial cells, belonging to the MUCl family [8]. Damaged alveolar epithelial cells release large amount of KL-6, which then can cause fibroblast aggregation and the inhibition of fibroblast apoptosis. When alveoli injured, the blood exchange barrier permeability increased, and the KL-6 swarm into the blood, which resulted in increased serum KL-6 level [19]. Given that the KL-6 is not an alveolar surfactant, over production of KL-6 may affect the expression of the original surfactant, alveolar expansion and then damage lung compliance, leading to ventilation dysfunction and long-term lung injury [20]. Therefore, the serum KL-6 level has been considered as a marker to predict the onset of ILD [21].

In our study, we found that the serum KL-6 level in CTD-ILD patients was significantly higher than all other groups, suggesting that the increased serum KL-6 level may indeed indicate the occurrence of ILD. Interestingly, there were a few patients presented high serum KL-6 level without obvious clinical symptoms or detectable pathological image by HRCT. It is possible that the alveolar structures of these patients sustained some damages, but not to a degree showing clinical and imaging manifestations, so future follow-ups will be required to discern if these patients will progress to ILD in the future, which can help us to further evaluate whether serum KL-6 level can be used to predict the onset of CTD-ILD before the appearance of clinical symptoms or any detectable changes by imaging exams.

The long-term use of corticosteroids and immunosuppressive agents in CTD patients increases the risk of pulmonary infection, which is a major cause of the mortality. Meanwhile, the rapid progression of CTD-ILD is often triggered by infection; so early identification of cough symptoms or lung infection caused by CTD-ILD is critical for guiding clinical treatment [22]. It has been shown that the level of serum KL-6 can be used to identify SLE (systemic lupus erythematosus) lung infection and SLE-ILD [23]. Interestingly, our study found that the level of serum KL-6 in patients with CTD-ILD is even higher than that of pulmonary infection, which would require further analyses to better understand its diagnostic value. Previous literatures reported that the level of serum KL-6 was significantly increased in patients with *Pneumocystis carinii* infection and showed no significant difference when compared with CTD-ILD patients. Because it is difficult to distinguish *Pneumocystis carinii* infection and ILD on HRCT alone, we should seek other etiological evidence in the clinic to help making accurate diagnosis and treatment [24]**.**

As for CTD-ILD patients, the CTX treatment plays a crucial role in their prognosis and is the only immunosuppressive agents proven by randomized controlled study that can effectively stabilize the patient’s lung function. Previous studies have suggested that the serum KL-6 levels can be used to evaluate the effectiveness of CTX pulse therapy for SSc-ILD patients [11, 15]. Consistently, data from our study showed that the level of serum KL-6 in CTD-ILD patients with improved condition after CTX treatment was significantly reduced (Figs. 3 and 4). Therefore, we believe that the changes in serum KL-6 levels may be used to assess the therapeutic effect of CTX pulse therapy in the CTD-ILD patients and their following prognosis. However, due to the low AUC of the ROC curve, it is prudent to use the measured KL-6 value as one of the indicators, preferably in combination with other existing biomarkers for the clinical evaluation on treatment effectiveness (Fig. 5).

Indeed, some limitations on the result interpretation existed in our study. For instance, we did not consider the effects of smoking and other possible confounding factors during our study, including age, gender, races, patient occupations and living condition/environments, due to the lack of access to larger sample sets from multiple geological locations, given the limited resource we currently have. In addition, with the small size of patient sample we collected and analyzed, as well as the heterogeneous nature of CTD-ILD diseases (which was inadvertently included due to limited patient recruitment period), it would be prudent to view our results as pilot data since several possible confounding factors, including distinct types of CTD, could affect or bias the result interpretation.

## Conclusions

From our analysis, we believe that the elevated serum KL-6 level should be suitable to be used to support the diagnosis of CTD-ILD disease, or at least as an auxiliary indicator. In addition, the reduction of serum KL-6 level after CTX pulse therapy presents itself as a supporting indicator for treatment effectiveness in the CTD-ILD patients in combination with other diagnostic biomarkers. Finally, even though our study was limited in scale, we remain confident that serum level of KL-6 could be a promising biomarker for the severity of interstitial lung disease in the future, which would undoubtedly depend on more detailed and comprehensive further studies to address confounding variables first.

## Data Availability

The datasets supporting the conclusions of this article are included within the article.

## Abbreviations

CTD: Connective tissue diseases
CTX: Cyclophosphamide
DLCo: Carbon monoxide diffusing capacity
ELISA: Enzyme linked immunosorbent assay
HRCT: High-resolution computed tomography
KL-6: Serum krebs von den lungen-6
PFT: Pulmonary function test
SSc: Systemic sclerosis
